# Very large-scale diffraction investigations enabled by a matrix-multiplication facilitated radial and azimuthal integration algorithm: *MatFRAIA*

**DOI:** 10.1107/S1600577522008232

**Published:** 2022-10-04

**Authors:** Alexander Bernthz Jensen, Thorbjørn Erik Køppen Christensen, Clemens Weninger, Henrik Birkedal

**Affiliations:** a Department of Chemistry and iNANO, Aarhus University, Gustav Wieds Vej 14, 8000 Aarhus, Denmark; b Sino-Danish College (SDC), University of Chinese Academy of Sciences, People’s Republic of China; c MAX IV Laboratory, Lund University, Lund, Sweden; ESRF and Université Grenoble Alpes, France

**Keywords:** azimuthal integration algorithms, data reduction, computer programs, scattering, 2D detectors, integration, data processing

## Abstract

As synchrotron facilities continue to generate increasingly brilliant X-rays and detector speeds increase, swift data reduction from the collected area detector images to more workable 1D diffractograms becomes of increasing importance. This work reports an integration algorithm that can integrate diffractograms in real time on modern laptops and can reach 10 kHz integration speeds on modern workstations using an efficient pixel-splitting and parallelization scheme. This algorithm is limited not by the computation of the integration itself but is rather bottlenecked by the speed of the data transfer to the processor, the data decompression and/or the saving of results. The algorithm and its implementation is described while the performance is investigated on 2D scanning X-ray diffraction/fluorescence data collected at the interface between an implant and forming bone.

## Abstract 2

A new integration algorithm for converting 2D diffractograms to azimuthally resolved diffraction patterns has been developed, and is able to carry out this function at kilohertz speed on modern hardware.

## 1. Introduction

The increased brilliance of synchrotron sources means that diffraction experiments on materials increasingly contain very large amounts of data. Such experiments include scanning or tomographic diffraction experiments. In a recent publication, for example, human bone was investigated by X-ray powder diffraction computed tomography (XRD-CT) (Wittig *et al.*, 2019 ▸). The raw dataset comprised over 2.6 million diffraction patterns, each measured on a large 2D detector with 4 million pixels. Other experiments involving large data volumes include SAXS/WAXS tensor tomography (Grünewald *et al.*, 2020 ▸). Optimized beamlines for XRD-CT and similar experiments have been designed (Vaughan *et al.*, 2020 ▸). As such experiments become more frequent (Palle *et al.*, 2020 ▸; Wittig *et al.*, 2019 ▸; Dong *et al.*, 2021 ▸; Jacques *et al.*, 2011 ▸; Vamvakeros *et al.*, 2018 ▸, 2020 ▸, 2021 ▸; Jensen *et al.*, 2021 ▸), the speed of integration, *i.e*. the transformation of the data from pixel coordinates to azimuthal and scattering vector length coordinates, becomes a critical component in the data analysis pipeline. This calls for highly efficient data integration methods. Two such data-treatment platforms are *PyFAI* (Kieffer *et al.*, 2018 ▸, 2020 ▸; Ashiotis *et al.*, 2015 ▸; Kieffer & Ashiotis, 2014 ▸; Kieffer & Karkoulis, 2013 ▸) and *SAXSDOG *(Burian *et al.*, 2020 ▸) which operate efficiently and with great flexibility. However, a need remains for even faster implementation of integration. Herein we describe further improvements to integration that are very efficient and in practice enable data reduction to be completed during synchrotron experiments.

## 2. Methods

We first describe the proposed algorithm and its implementation followed by a description of a test experiment.

### 2.1. Matrix-multiplication facilitated radial and azimuthal integration algorithm

Integration of 2D XRD data from detector pixel coordinates into polar coordinates [scattering angle 2θ (or scattering vector length *q*) and azimuthal angle χ] involves two steps: a coordinate transformation and subsequent summation of weighted contributions to each 2θ,χ bin. Done directly with an indexing matrix is very inefficient. The core concept in the matrix-multiplication facilitated radial and azimuthal integration algorithm (*MatFRAIA*) is to conduct these operations by indexing using sparse matrices (our implementation uses a compressed sparse row on the GPU and a compressed sparse column on the CPU to comply with cuBLAS and BLAS, respectively). Prior to integration, an indexing matrix is calculated that contains the location in 2θ,χ space of each pixel as well as the weight with which it contributes, defined as its relative area. Once this indexing matrix is at hand, integration is a matter of simple matrix multiplication between the index matrix and the current detector frame, which is a linear transformation.

#### 2.1.1. Pixel splitting

**Figure 1:**
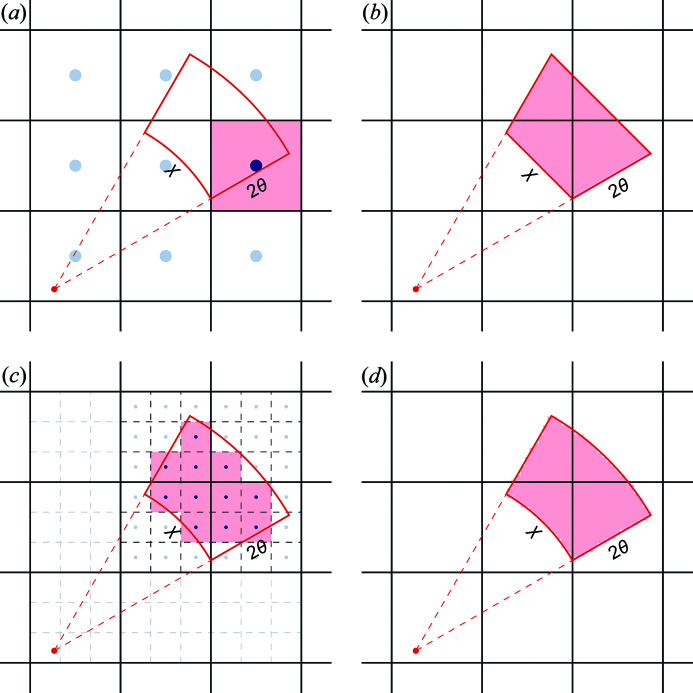
Pixel splitting improves assignment of areas of the detector into the appropriate (2θ,χ) bin during integration. (*a*) No splitting. Here only the pixels with their center within a (2θ,χ) bin are assigned entirely to said bin. (*b*) Method employed by *PyFAI*. Here the analytical area of the trapeze spanned by the azimuthal lines fall into the bin, yet it does not take into account the curvature of the arc. Pixel splitting by super-sampling (*c*) 3 or (*d*) 20 times in both cardinal directions. This is the method employed by *MatFRAIA*. The red area shows which part of the detector will be included in the resulting (2θ,χ) bin.

Integration involves a transformation from Cartesian detector coordinates to polar coordinates (2θ,χ), where 2θ is the radial coordinate expressed as the scattering angle and χ is the azimuthal angle around the direct beam. Due to the finite area of detector pixels, their effective weight in (2θ,χ) space varies with location on the detector with pixels close to the beam center covering a larger area in (2θ,χ) than pixels far from the center. Similarly, pixels along the diagonal (χ = 45°, 135°, 225°, 315°) are longer in the 2θ direction than pixels along the cardinal directions (χ = 0°, 90°, 180°, 270°). To ensure effective attribution of intensity, it has been proposed that pixel splitting should be used, where each detector pixel is virtually split, either to fit with the resulting bins, as done by *PyFAI* (Kieffer & Ashiotis, 2014 ▸), or by splitting the pixel into an arbitrarily fine set of sub-pixels chosen to reduce the nonlinear transformation effects while not being too computationally heavy. *MatFRAIA* adopts the latter approach, which is especially important for azimuthally resolved analyses. One way to deal with this problem is to virtually split detector pixels to allow for a more accurate account of their contributions to a given (2θ,χ) bin in the integrated data. There have been multiple suggested methods of such pixel splitting. In Fig. 1 ▸, we illustrate some of these. With no splitting at all, only pixels that have their centers within the final bin will fall into said bin. This method describes the bins poorly as seen in Fig. 1 ▸(*a*), unless very large bins are used. One possible solution is that employed by *PyFAI* as full splitting assuming straight bin edges (van der Walt & Herbst, 2007 ▸, 2012 ▸; Ashiotis *et al.*, 2015 ▸), which is illustrated in Fig. 1 ▸(*b*). It approximates the χ-dependent curvature of the bins to straight lines, which is not fully accurate. Another approach is super-sampling the detector frame; this is employed in both *MatFRAIA* and *SAXSDOG* (Burian *et al.*, 2020 ▸). This approach is illustrated with super-sampling each pixel either 9 (3 × 3) or 400 (20 × 20) times in Figs. 1 ▸(*c*) and 1(*d*), respectively. This approach provides means of systematically reducing the curvature problem and with infinite, but in practice much lower, splitting resulting in a perfect transformation. Initially the idea of super-sampling each pixel say 400 times might seem too computationally heavy, but it only needs to be performed once for each setup (*i.e.* calibration and integration settings). After that, a linear transformation from pixel space to (θ,χ) space is computationally efficient and fast. Storing this transform as a sparse matrix makes the integration both efficient and heavily reduces its memory footprint.

The index matrix is typically sparse and significant memory savings are obtained by employing sparse data representations. This use of sparse matrices is similar to the procedure used by *PyFAI*; however, since the data are super-sampled in a grid, geometrical corrections such as the Lorentz, polarization or solid-angle correction can be applied on a subpixel level instead of on a pixel level, and this can be incorporated directly into the integration transformation.

**Figure 2:**
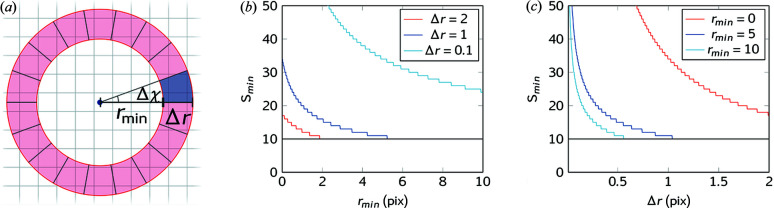
Minimum splitting factor. (*a*) Splitting area illustration. The blue dot shows the beam center, the red shaded area corresponds to the 1D 2θ bin with a full integration and the blue shaded area corresponds to a possible (2θ,χ) resolved bin with Δχ = *N*_Ab_/2π. (*b*, *c*) Graphs of the minimum pixel-splitting curve with *N*_Ab_ = 360 and ρ_sp_ = 5 as a function of (*b*) *r*_min_ and (*c*) Δ*r*.

The required level of pixel splitting has previously been suggested to be rather low; the *SAXSDOG* (Burian *et al.*, 2020 ▸) documentation suggests that going above *S* = 3 is ‘overkill’ whereas the *DIOPTAS* program (Prescher & Prakapenka, 2015 ▸) has the feature disabled by default. We suggest that much larger scaling is needed depending on the end goal of the integration, *i.e.* the number of output bins in (2θ,χ), especially if χ-resolved analyses are required. To estimate the required level of pixel splitting, the effective area of the pixels is needed. They can be described using four factors: (1) the number of azimuthal bins, *N*_Ab_; (2) the number of sub-pixels desired in each (2θ,χ) bin, ρ_sp_, which is effectively a pixel-splitting precision measure; (3) the relation of this area to the smallest effective detector area (in pixels), given by the smallest radial distance that one wishes to include (corresponding to the smallest value of 2θ), *r*_min_; and (4) the effective Δ2θ, which is given by the largest radial bin width, Δ*r*, measured in pixels. This is illustrated in Fig. 2 ▸(*a*). To get an estimated appropriate pixel-splitting number, the square root of the area ratio is needed. To find the required pixel splitting the following formula can then be used:

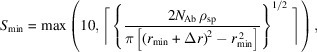

where we suggest a minimum splitting of 10 for azimuthally resolved integration, *i.e.* a minimum splitting of each pixel into 100 sub-pixels. Splitting pixels into 100 sub-pixels is sufficient to remove the effects of the diagonal pixels being longer in the 2θ direction, but it might not always be enough to reach the desired statistics in the final bins, thus sometimes a larger splitting is required. Figs. 2 ▸(*b*)–2(*c*) show examples of how the size of 2θ bins (Δ*r*) and the minimum 2θ values (*r*_min_) influence *S*_min_. For most real-world scenarios, our experience suggests that a splitting factor of 20–30 (splitting each pixel into 400–900 sub-pixels) is plenty. Splitting by these larger factors greatly increases the calculation time and memory footprint of the indexing matrix, as they both scale with *O*(*S* ^2^); however, in the next section, we also show how to reduce the memory footprint during the calculation of *O*(*S*). We further stress that the choice of the degree of pixel splitting is strongly influenced by the choice of *r*_min_, hence, in many cases of diffraction analyses, smaller pixel splittings can be used. The level of pixel splitting is thus a parameter provided to the algorithm by the experimenter.

#### 2.1.2. MatFRAIA

The *MatFRAIA* algorithm involves the following steps assuming the detector center, tilt and sample-to-detector distance have been obtained by calibration. The *MatFRAIA* *MATLAB* scripts include a subfunction for reading PONI files to represent the point of normal incidence rather than the detector center and tilt. In the following, we assume that a number of frames were collected and organized into files containing a number of individual frames. The algorithm is split into two parts, creating the integration transform and applying the integration transform to the data.

(1) The indexing matrix is built given a chosen pixel splitting, *S*, and bin sizes in 2θ,χ spaces:

(*a*) To reduce the memory footprint, the indexing matrix is built in sections. Thus a matrix sized by a subsection of the detector frame of size 1/*S* of the detector frame is allocated, our implementation uses vertical slices going from the left to the right of the detector, then this image is rescaled *S* × *S* times the original size, splitting all pixels into *S* × *S* sub-pixels.

(*b*) Tilt, polarization correction and Lorentz corrections are applied.

(*c*) Linearized pixel indices are calculated: a pixel is labelled using *m*, *n* and a linear index is calculated by *i* = (*m* − 1)*n*_max_ + *n*, where *n*_max_ is the largest value of *n*. The same linearization is used for the radial (2θ) and azimuthal (χ) indexes to give the polar coordinate index vector **j**. The indexing matrix is then spanned by the index vectors **j** and **i** with the (*j*, *i*) entry in the matrix being the weight, *w*, of the *i*th detector pixel in the *j*th 2θ,χ bin. This matrix is effectively sparse, which strongly reduces the memory requirements when saved in a compressed format. *w* values for (*j*, *i*) are computed from the pixel-splitting image.

(*d*) Steps (*a*)–(*c*) are repeated *s* times, appending the sparse matrices.

(*e*) Any required masks are applied, *i.e.* corrections on all the weights in *i* that point on the same *j* where some *i* should be masked out (Zingers, beam stop or detector units).

(*f*) The indexing matrix *A* is now of size (*j*_max_, *i*_max_) or (2θ_max_χ_max_, *n*_max_ *m*_max_). To further reduce its memory footprint and processing time, all bins that are masked out due to insufficient number of unmasked-subpixels going into the bin are all removed except for the first entry (*j*, *i* = 1), which is set to NaN (not a number), resulting in the *j*th output bin being NaN.

(2) For parallelization purposes one worker for every thread of a processor can work on a given file simultaneously; this will however increase memory consumption. To work around this, each worker can load a subset of its file, keeping the total memory consumption constant. Parallelization is done on a file-by-file basis, in order to optimally utilize the connection to the storage drive/server, as well as to allow parallel decompression, even when using deprecated compression algorithms:

(*a*) A part of the datafile is loaded, *e.g.* *d* detector frames (*d* < 20 is common for parallelization work).

(*b*) Hot pixels/accidental high-intensity signals not caught in the original mask are temporarily removed.

(*c*) The data matrix *B* is reshaped to shape (*i*_max_, *d*) so the indexing matrix can be applied.

(*d*) The integrated data in 2θ,χ space is calculated by applying the indexing matrix to the detector frame [*i.e.* matrix multiplication; *C* = *AB* has the shape (*j*_max_, *d*)].

(*e*) *C* is reshaped to size (2θ_max_, χ_max_, *d*).

(*f*) The first *d* integrated data frames are saved.

(*g*) Steps (*a*)–(*e*) are repeated until the file has been fully integrated.

(*h*) Steps (*a*)–(*f*) are repeated for every datafile.

Before integration the detector frames typically need to be masked, *e.g.* due to dead pixels, detector mount, beam stop shadow *etc*. This is achieved by creating a logical matrix, where all data pixels are marked as ‘true’ and all non-data are marked as ‘false’. This mask is given to the indexing algorithm.

We implemented *MatFRAIA* in both *MATLAB* and Python. The benchmarking performed herein was conducted on the *MATLAB* version; a similar performance was observed with the Python version. The *MATLAB* version of the algorithm is available at https://gitlab.au.dk/hb-group/matfraia. The Python version of the algorithm is available at https://github.com/maxiv-science/azint.

#### 2.1.3. Performance benchmarking

The performance of the *MatFRAIA* code was tested both on a standard laptop running Debian Linux with an Intel Core i7-8550U (4 cores and 8 threads running at 1.8–4 GHz) with 16 GB of RAM as well as on a workstation running Windows with an AMD EPYC 7502p (32 cores and 64 threads processor running at 2.5–3.35 GHz) and equipped with 512 GB RAM. These performance tests were carried out using a dataset consisting of 38152 detector frames across 340 lz4-compressed hdf5-formated files taking up 150 GB of disk space. We note that a more recent compression scheme is available, bitshuffle (Masui *et al.*, 2015 ▸), which allows for parallel decompression. However, our algorithm decompresses multiple files in parallel, so the performance should be portable between the compression schemes.

On the laptop, the data were loaded both from a server over a 1 GB s^−1^ connection and from an HDD over a 1.6 GB s^−1^ USB-3 connection. On the workstation, the data were loaded from a server over a 1 GB s^−1^ connection as well as with the datafiles saved onto a local 300 GB RAM-disk to examine efficiency when file transfer/loading is as unlimited as it can be for this amount of data. We measured the transfer speed on this RAM-disk to be ∼1.9 GiB s^−1^.

While the indexing time changes with increased pixel splitting, the integration time does not change significantly, thus benchmarking the effect of different pixel-splitting factors was only conducted for the indexing with splitting *S* = 20. Changing the number of output bins in the integration will change the integration time slightly.

For the benchmarking, the code ran for 1 min without performing any calculations to find the baseline system memory usage. The average of this level was then subtracted to set the zero-level memory consumption before *MatFRAIA* started calculations.

### 2.2. Experimental

We collected scanning XRD and X-ray fluorescence (XRF) data on a test sample consisting of a grade 4 Ti implant that had been inserted into rat knees. After 4 weeks of healing time the animals were euthanised, the bone was then cut into thin slices using a diamond saw (Accutom-5, M1D18 blade, Struers, Ballerup, Denmark). The samples were then further polished using increasingly finer abrasive paper (Struers, P320–P4000) to a final thickness of 327 µm; the samples were then ready to scan. This was part of a larger ongoing study on the structural changes around implants that will be reported elsewhere. The data were used to benchmark the performance of *MatFRAIA*. The implant had a spring inserted to give tension near the screw head, and the bone that had grown around the tension spring is used as the test subject here.

#### 2.2.1. Scanning XRF/XRD data collection

Data were collected at beamline P06 at the PETRA III synchrotron (DESY, Hamburg) (Schroer *et al.*, 2010 ▸). An X-ray energy of 17 keV was used. The beam was focused by Kirkpatrick–Baez mirrors (Kirkpatrick & Baez, 1948 ▸) to a beam size of 450 nm × 370 nm. The sample was scanned using continuous scans in steps of 1 µm with exposure times of 0.05 s for each step. XRF was detected by a Vortex-EM silicon drift detector (Hitachi, USA), the spectra were fitted using a *PyMca* (Solé *et al.*, 2007 ▸) fitting core along with an in-house multi-threaded fitting wrapper. The diffraction data were collected using a DECTRIS EIGER X 4M detector. A total of 38152 points were measured in a grid measuring 110 µm × 350 µm over a period of 36 min and 33 s resulting in a data acquisition rate of 17.4 Hz. Treatment of the XRD data was conducted with *MatFRAIA* while the XRF data were used for reference and were plotted using in-house *MATLAB* scripts.

#### 2.2.2. Orientation analysis

**Figure 3:**
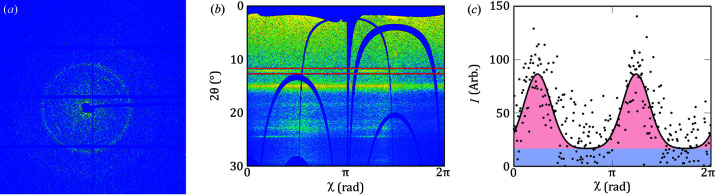
Example of test data. (*a*) Raw detector frame. (*b*) Azimuthally resolved detector frame after *MatFRAIA* integration, with the HAP (002) peak marked. (*c*) Azimuthally resolved HAP (002) intensity after local background subtraction. Data are shown as black dots, the Gaussian fit is shown as a black line, and the amount of oriented and non-oriented crystallites are indicated in red and blue areas, respectively.

In the present example, we analyzed the orientation of hy­droxy­apatite (HAP) biomineral nanocrystals projected across the sample. First, the raw diffractograms, an example of which is shown in Fig. 3 ▸(*a*), were integrated with the *MatFRAIA* algorithm as described and the data kept were azimuthally resolved. Once the detector frames were integrated, the integration transformation turned them into (2θ, χ)-resolved images such as that shown in Fig. 3 ▸(*b*) that was obtained by applying the integration transformation to the diffractogram in Fig. 3 ▸(*a*). From here, a peak of interest can be selected. In bone, the HAP crystallites are often preferentially oriented along the crystallographic *c* direction, thus the (002) peak, indicated in Fig. 3 ▸(*b*), was chosen for orientation analysis. The background was then subtracted from the selected peak before it was integrated over 2θ. Subsequently, the orientation was determined using custom-written *MATLAB* code with Gaussian fits to the azimuthal intensity dependency (Rinnerthaler *et al.*, 1999 ▸; Wagermaier *et al.*, 2007 ▸),

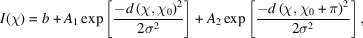

where *A*_1_ and *A*_2_ are the intensities of the two peaks, which are not necessarily equal due to the asymmetric intersection of the Ewald sphere. To place the two azimuthal peaks on a circle [and thus circumvent potential truncation effects more easily compared with the more standard distance function (χ, χ_0_) = χ − χ_0_ (Bünger *et al.*, 2010 ▸; Rinnerthaler *et al.*, 1999 ▸)] we write the position function as

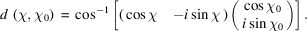

This approach was used to fit the azimuthal intensity of the (002) HAP peak. Such a fit is shown in Fig. 3 ▸(*c*). From the fit the orientation of the crystals is given by χ_0_ and the projected degree of orientation (DoO) is given by

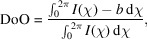

that is, the proportion of the peak intensity in the point stemming from oriented crystals to the total signal in the point. Information is also gained about the density of oriented crystals

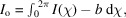

the density of randomly oriented crystals

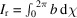

and the total density of crystals at the point

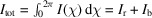

(note that crystals with the *c* axis parallel to the direct beam are effectively invisible and thus this method is only sensitive to the manifold of crystals that are observable in the given projection).

## 3. Results

The *MatFRAIA* algorithm affords rapid integration of large volumes of data to the point that it can be used for online processing of data during synchrotron beam time, see Section 3.1. It has been implemented in both *MATLAB* and Python. The method requires calculation of an indexing matrix, which is done once during a data processing step. The pixel splitting required depends on different factors, *e.g.* the number of output azimuthal bins. For full azimuthal integration into a 1D diffraction pattern, a lower degree of pixel splitting is sufficient, whereas, for full azimuthal analysis, a larger degree is needed. In our experience, a pixel splitting of 20 produces data without fail for 360 χ bins for a DECTRIS X 4M detector.

### 3.1. Performance evaluation

The results of the speed test are summarized in Table 1 ▸. During the test the following serial (*i.e.* the actual physical time spent on the process) times were measured: the total time for the algorithm, the indexing (the first step of the algorithm) and the execution (the second step of the algorithm). As the integration is carried out in parallel, each worker (or thread) can also report on time spent on sub-processes during integration, thus each worker reports how much time is spent on loading and decompressing the data, on pre-processing the data for integration, on integrating the data, on saving the integrated data, on overhead, and how much time is spent in total. The algorithm is quite fast and most time is in fact spent loading and decompressing data, except when the data are stored on a RAM-disk, eliminating most of the loading time. Comparing results from the laptop (first and second lines in Table 1 ▸) we can see that even a laptop can saturate a 1 GB ethernet connection, and that the process becomes slightly faster using a local hard disk. On the workstation, we compared a 1 GB net connection with having data preloaded onto a RAM-disk to minimize loading time. Even in the RAM-disk case, the loading/decompression time mainly consists of load times, as we read the data at 1.876 GiB s^−1^. The results show that the algorithm can integrate anywhere from 4 to 10 kHz on the workstation [depending on whether RAM-disk (5.63% of 160.16 s spent integrating 38152 frames or 4.2 kHz) or ethernet 1 GB s^−1^ (0.26% of 1438.03 s spent integrating 38152 frames or 10 kHz) is used, Table 1 ▸]. However, the laptop achieves 500–641 Hz in ‘integration time alone’ (4.30% of 1384 s spent integrating 38152 frames or 641 Hz for 1 GB s^−1^, or 6.35% of 1197.5 s spent integrating 38152 frames or 502 Hz), which should be sufficient for the data stream at most beamlines. However, these integration frequencies are for the integration itself. Real-world data are compressed and saved prior to integration. Even so, the algorithm can still keep up on a 1 GB s^−1^ connection with an integration rate above 17.4 Hz, the rate at which the data were measured, on the laptop, while achieving more than 229 Hz on the workstation. The rate of 17.4 Hz corresponds to 125 MB s^−1^, which is well above what was reported in *SAXSDOG* (Burian *et al.*, 2020 ▸). We reiterate that on both the laptop and the workstation most of the time is spent loading, decompressing and rearranging the data, as shown in Table 1 ▸. Indeed, it is important to differentiate between the speed of the actual integration itself and the total time taken by all the necessary steps of the algorithm on compressed data. It is evident from Table 1 ▸ that the actual integration time becomes small compared with the overhead of loading and reshaping data. For all the data storages demonstrated, the integration is limited by loading the data, even for the RAM-disk on the workstation, where the transfer speed reached was approximately 1.875 GB s^−1^, which is as fast as the RAM-disk with a file system would allow data to be transferred.

In our experience, many current scanning-type or *in situ*-type measurements do not often exceed speeds on the order of 100 Hz. The present algorithm can thus keep up with realistic data collection speeds for a vast range of experiments.

**Figure 4:**
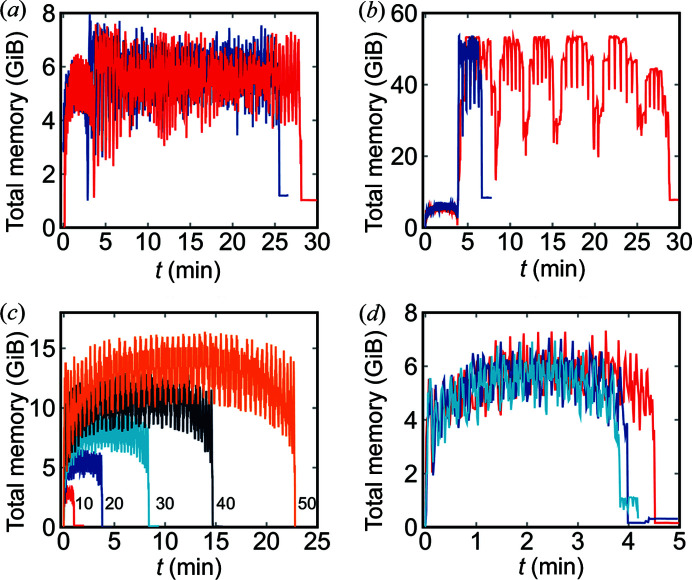
Memory footprint of *MatFRAIA*. (*a*) Memory footprint during integration and indexing on the laptop over ethernet (red) and from an HDD (blue). (*b*) Memory footprint during integration and indexing on a workstation over ethernet (red) and from a RAM-disk (blue). (*c*) Memory footprint during indexing with different pixel splittings. (*d*) Memory footprint during indexing with a different number of azimuthal bins *N*_ab_ = [1, 18, 360] for a pixel-splitting level of *S* = 20.

Aside from the integration time, another important factor for integration in a concrete experiment is the memory footprint. This is shown in Figs. 4 ▸(*a*) and 4(*b*) for integration performed on a laptop and a workstation, respectively. The first part of the curve shows the calculation of the indexing matrix used to perform the integration as a transformation with a pixel-splitting factor of 20 (400 subpixels per pixel). This part of the curve takes up approximately 6 GiB of RAM for less than 4 min, which might seem like a long time spent on making the indexing matrix; it is however necessary in order to get such a high level of pixel splitting. With a lower level of splitting, say *S* = 1, 3 and 9, the indexing matrix can be calculated in 1.14 s, 5.28 s and 38.87 s, respectively. The second part of the curve is the actual integration. Here the memory footprint is proportional to the number of workers (threads) used, and the time is proportional to the data transfer speed. In these tests, since they were carried out on compressed data, a significant amount of time was spent on decompression, even when loading had been mostly eliminated by storing the data on the RAM-disk. The algorithm will allocate the memory needed during integration; this is the reason the two curves in Fig. 4 ▸(*b*) have the same maximum memory footprint.

Though the memory footprint and computation time of the indexing increase with the pixel-splitting factor, as seen from Fig. 4 ▸(*c*), it increases much less than linearly with the number of azimuthal bins, as seen in Fig. 4 ▸(*d*). The main reason for the slight increase with the number of azimuthal bins is that the transformation matrix will be larger.

### 3.2. Online data treatment during measurements

The high speed of *MatFRAIA* makes it a good candidate for online data treatment, *i.e.* during data collection in synchrotron experiments. We tested the capabilities of *MatFRAIA* to be used as such in scanning XRF/XRD measurements at the PETRA III beamline P06 (see Experimental[Sec sec2]). As a result, the data were already integrated and ready for further analysis before we left the beamline, reducing what needs to be done after returning from an experiment. Furthermore, a Python implementation of *MatFRAIA* has been used at NanoMax at MAX IV (Björling *et al.*, 2021 ▸).

### 3.3. Example data: orientation analysis of bone around an implant

**Figure 5:**
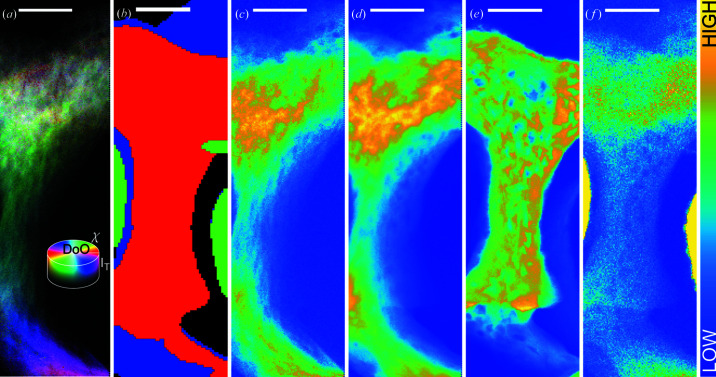
Analysis of the test sample consisting of a tensile load screw and surrounding bone. (*a*) Orientational distribution H(ue) S(aturation) V(alue) map of the full sample, for every pixel the process like that described in Section 2.2.2 has been carried out if sufficient HAP (002) signal was present. The hue of a point reveals the crystallite orientation, the saturation reveals the projected degree of orientation and the value reveals the total projected (002) intensity in the point. (*b*)–(*f*) XRF and XRD analysis. (*b*) Segmentation sketch based on XRF signals. The Ca signal in red shows areas where bone is close to the sample surface, the Ni signal in green shows the location of the implant tension spring, and the Sr signal in blue shows areas where there is bone further from the sample surface. (*c*) HAP (002) integrated intensity. (*d*) HAP multiplet integrated intensity. (*e*) XRF Ca signal. (*f*) XRF Sr signal. Scale bars are 50 µm. The outermost 3 µm in (*a*)–(*f*) have been removed on both the left and the right, due to edge effects; thus (*a*), (*c*) and (*d*) contain 104 × 350 diffractograms.

The bone test sample was investigated through position-resolved XRD/XRF analysis. A total of 38152 raw diffractograms were collected. The final dataset (Fig. 5 ▸) was truncated to 36400 raw diffractograms [such as the one shown in Fig. 3 ▸(*a*)] to account for scan-edge effects.

The data were assigned to multiple sections corresponding to different material contributions – bone, implant tension screw and nothing – as shown in Fig.5 ▸(*b*) ▸, which is a segmentation based on Ca (red), Ni (green) and Sr (blue) XRF signals. The red segment corresponds to bone near the surface of the sample oriented towards the XRF detector and is based on the Ca signal in Fig. 5 ▸(*e*) ▸. The blue signal in Fig. 5(*b*) ▸ corresponds to bone deeper in the sample and is based on the Sr signal in Fig. 5 ▸(*f*) ▸. The difference between the Ca- and Sr-based bone assignment originates from the rather high thickness of the sample, meaning that the Ca signal only reports on the part of the sample close to the surface whereas the Sr signal reports on the full thickness since Sr *K*α is higher in energy and thus can escape from the full depth of the sample. Lastly the green signal in Fig. 5 ▸(*b*) ▸ corresponds to the tension spring in the sample around which the bone has grown and is based on the Ni XRF signal. As seen in Fig. 5 ▸(*f*) ▸ enough Ni signal can pile up and give rise to what seems to be Sr signal. As the diffraction signal has the highest energy, it carries all the way through the sample, thus the HAP multiplet [HAP (211), (112), (300) and (202) peaks located around 2θ = 15° at this experiment energy, see Fig. 3 ▸(*b*)], as shown in Fig. 5 ▸(*d*) ▸, shows the full outline of the bone. The integrated HAP (002) peak, as shown in Fig. 5 ▸(*c*) ▸, is not visible in all areas due to orientation effects. Based on the HAP (002) peak, the orientation of bone in all points can be found, as described in the section *Methods* [Sec sec2].

The results of the orientation analysis are summarized in Fig. 5 ▸(*a*), ▸ which reports the orientation as the color (hue) in each pixel while the degree of orientation is given as the saturation (white to colored) and the total (002) diffraction intensity is reported as the value (black to white). From here it can be seen that the bone has grown such that its crystals curl around the spring wire from the bottom of the region of interest all the way to the top. Farther from the spring, the mineral is randomly oriented in projection resulting in a low projected degree of orientation.

## 4. Discussion

The present implementation shares some similarities with the methods used in *PyFAI* that also uses pixel splitting and indexing. In *PyFAI* the indices are implemented through a sparse matrix and/or a lookup table (Kieffer & Ashiotis, 2014 ▸) akin to the current index matrix. The present version of a linearized index allows very efficient use of sparse matrix multiplication in *MATLAB*, which is at the core of the performance obtained herein. Similar performance was found in a Python implementation.

The *MatFRAIA* pixel-splitting scheme allows for certain effects to be applied to a sub-pixel level, which might be desirable; it is also, to the best of our knowledge, the first pixel-splitting scheme that allows for an arbitrary accuracy while conforming to the integration bin shape.

The present *MatFRAIA* implementation utilizes parallelization on a per-file basis. Though this might not be important for smaller datasets, it allows the data to be not only integrated but also loaded in parallel, which increases the chance of the data integration process fully utilizing the bandwidth to the data location.

## 5. Conclusions

We have presented the new *MatFRAIA* integration algorithm utilizing both a novel pixel splitting scheme and a novel parallelization scheme while incorporating techniques akin to those used in *PyFAI*, namely using a sparse indexing matrix for the integration itself. Using *MatFRAIA* we have shown integration speeds of multiple kilohertz to be possible using modern hardware, and that a common laptop can saturate its own ethernet connection, while being able to keep up with the pace at which data are gathered at experiments on current synchrotrons. Furthermore, we have shown that the actual integration is now quite fast, suggesting that further improvements could arise from gaining efficiency in other parts of the process, such as compression and structuring the data for integration, or by implementing on-board integration in parallel to compression of detector frames.

## Figures and Tables

**Table 1:** Benchmarking timings of the algorithm for different real-world scenarios Discrepancies between ‘Execution time’ and ‘Time spent in job’ can be attributed to parallelization overhead.

Measure	Laptop (1 GB s^−1^)	Laptop HDD (1.6 GB s^−1^)	Workstation (1 GB s^−1^)	Workstation (RAM-disk)
Total time (s)	2124	1648	1772	402
Indexing time (s)	227	201	229	228
Execution time (s)	1897	1446	1510	166
Time spent in job (s)	1384	1197.5	1438.03	160.16
Loading and decompression time per worker (%)	77.35	61.36	96.52	49.91
Data processing time per worker (%)	14.73	30.84	1.69	44.14
Integration time per worker (%)	4.30	6.35	0.26	5.63
Save time per worker (%)	0.02	1.09	0.01	0.21
Overhead in job (%)	3.60	0.36	1.52	0.11
